# Connectome-based predictive modeling for functional recovery of acute ischemic stroke

**DOI:** 10.1016/j.nicl.2023.103369

**Published:** 2023-03-08

**Authors:** Syu-Jyun Peng, Yu-Wei Chen, Andrew Hung, Kuo-Wei Wang, Jang-Zern Tsai

**Affiliations:** a Professional Master Program in Artificial Intelligence in Medicine, College of Medicine, Taipei Medical University, Taipei, Taiwan; b Department of Neurology, Landseed International Hospital, Taoyuan, Taiwan; c Department of Neurology, National Taiwan University Hospital, Taipei, Taiwan; d Department of Electrical Engineering, National Central University, Taoyuan, Taiwan; e Department of General Affairs, Landseed International Hospital, Taoyuan, Taiwan

**Keywords:** Stroke, Connectome, Prediction, Functional recovery, Resting-state functional MRI, mRS, BI

## Abstract

Patients of acute ischemic stroke possess considerable chance of recovery of various levels in the first several weeks after stroke onset. Prognosis of functional recovery is important for decision-making in poststroke patient care and placement. Poststroke functional recovery has conventionally been based on demographic and clinical variables such as age, gender, and severity of stroke impairment. On the other hand, the concept of connectome has become a basis of interpreting the functional impairment and recovery of stroke patients. In this research, the connectome-based predictive modeling was used to provide predictive models for prognosing poststroke functional recovery. Predictive models were developed to use the brain connectivity at stroke onset to predict functional assessment scores at one or three months later, or to use the brain connectivity one-month poststroke to predict functional assessment scores at three months after stroke onset. The brain connectivity was computed from the resting-state fMRI signals. The functional assessment scores used in this research included modified Rankin Scale (mRS) and Barthel Index (BI). This research found significant models that used the brain connectivity at onset to predict the mRS one-month poststroke and to predict the BI three-month poststroke for patients with supratentorial infarction, as well as predictive models that used the brain connectivity one-month poststroke to predict the mRS three-month poststroke for patients with supratentorial infarction in the right hemisphere. The connectome-based predictive modeling could provide clinical value in prognosis of acute ischemic stroke.

## Highlights

• Using functional connectivity to predict poststroke functional recovery.
• Connectome-based predictive models consisting of significant edges.
• Evidence-based modeling that can be automated easily.

## 1 Introduction

Brain function is thought to be effected by the functional brain networks. Stroke impairs the physiology of the functional brain networks and leads to dysfunction of motor control, sensation, language, thinking, memory, etc. Poststroke treatment such as rehabilitation can help the patient to relearn the skills impaired due to the stroke. Through focused repetitive practice of a lost skill, the functional brain networks may undergo a circuit rewiring, so called neuroplasticity, to undertake the lost function. The effectiveness of functional recovery is assessed with various rating scores. For example, modified Rankin Scale (mRS) (Rankin, 1957) measures the degree of disability or the degree of dependence in daily activities. An mRS evaluation will result in one among the 7 scores ranging from score 0 for no symptoms at all to score 6 for being dead. Scores 1, 2, 3, 4, and 5 denote no significant, slight, moderate, moderately severe, and severe disability, respectively (Broderick et al., 2017). For another example, Barthel Index (BI) is used to assess a patient’s progress toward independence by evaluating the physical assistance required by the patient to perform activities (Mahoney, 1965).

Timing is a crucial factor for a successful recovery. An earlier treatment will generally result in a better recovery of the impaired skills. A prognosis in the acute stage helps to direct the decision of poststroke medical treatment or rehabilitation on the basis of individual recovery potential (Koch, 2021). However, prediction of stroke outcomes remains a clinical challenge despite numerous prediction tools have been developed (Stinear et al., 2019). For example, a multivariate linear regression model was developed for predicting BI score based on the patient age, sex, binarized Glasgow Coma Scale score, National Institutes of Health Stroke Scale (NIHSS) score, and stroke subtype (Douiri, 2017).

The connectome has recently been used to develop prediction models for researches of attention (Yoo, 2018), cocaine abstinence (Yip, 2019), anxiety (Wang, 2021), propensity (Lu, 2019), loneliness (Feng, 2019), etc. A connectome-based predictive model (CPM) (Shen, et al., 2017) is built by selecting from the whole-brain data a set of interregional functional connections most relevant to the predicted target and using these selected as the predictors. There have been different definitions of function connectivity based on various data modalities (Eickhoff, 2015). In this research, the functional connectivity of two brain regions refers to the temporal correlation of their blood oxygenation level-dependent (BOLD) signals assessed with resting-state functional MRI (rs-fMRI).

Depending on the number of brain regions of interest, there may be tens of thousands or even more functional connections in a whole brain. Among such a huge amount of functional connects, usually only a few are associated with the target parameter to be predicted (feature selection). To build a connectome-based predictive model of stroke outcome, a large amount of data must be provided to train the model. In the training process, a set of the most relevant functional connects will be identified to form a predictor of the CPM. The selection of functional connects and the model building of CPM is conceptually similar to the training process of machine learning of artificial neural network.

This research was aimed at devising a procedure of connectome-based predictive model for prognosis after acute ischemic stroke. Specifically, it would be interesting to investigate whether a predictive model can be derived solely based on the inter-regional connectivity.

## 2 Method

### 2.1 Subject recruiting

**Fig. 1:**
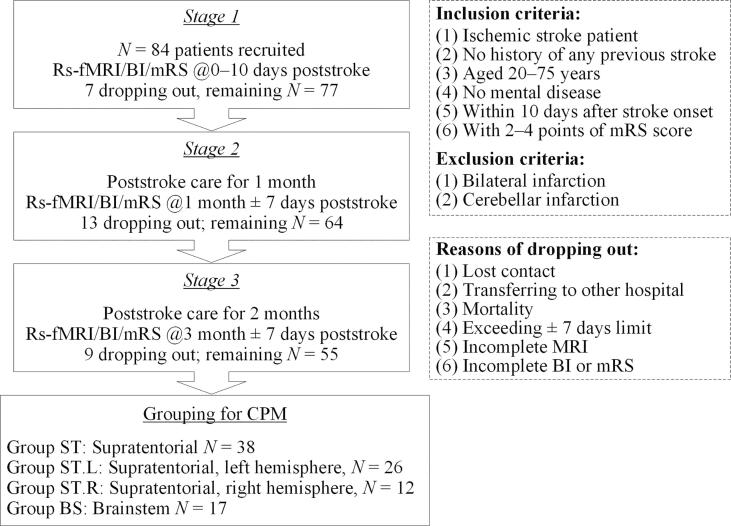
The three stages of MRI acquisition and functional assessment, followed by patient grouping for conducting connectome-based predictive modeling (CPM).

Acute ischemic stroke patients admitted to Landseed International Hospital, Taoyuan, Taiwan in 10 days of onset were recruited in the study. The study period was between Jan. 2015 and Dec. 2018. The inclusion and exclusion criteria are shown in Fig. 1. Each patient recruited and his/her legal representatives were informed with the motivation and the aim of this research and the right of the recruited before a consent was signed by the legal representatives. The recruited patients received routine poststroke medical care including rehabilitation. As depicted in Fig. 1, the levels of disability and dependence were assessed with mRS and BI along with MRI acquisition at three stages—the day of stroke onset, one month after onset, and three months after onset. The collected MR images included T1-weighted (T1w) images to be used for defining anatomical regions of interest (ROI), diffusion-weighted images (DWI) for clinical inspect of infarct locations, and rs-fMRI for derivation of functional connectivity. The protocol of this research has been reviewed by the Institutional Review Board (IRB) of the hospital.

### 2.2 MRI acquisition

The radiological imaging data were acquired in the Radiology Department of Landseed International Hospital, Taoyuan, Taiwan, with a 1.5-T Signa HDxt (GE Healthcare, Milwaukee, WI, USA).

The rs-fMRI data were obtained by scanning using an echo planar imaging sequence. The number of axial slices = 25, repetition time (TR) = 2000 ms, echo time (TE) = 35 ms, flip angle (FA) = 90°, slice thickness = 5 mm, inter-slice gap = 6 mm, field of view (FOV) = 220 × 220 mm^2^, matrix size = 64 × 64, the duration of each scan session = 330 s.

The 3D T1w images were acquired using three-dimensional magnetization-prepared rapid gradient-echo (3D MPRAGE) imaging with the following parameters: matrix size = 256 × 256, number of axial slices = 120, voxel size = 0.976 × 0.976 × 1.2 mm^3^, spacing between slices = 1.2 mm, inversion time (TI) = 450 ms, TR = 9.956 ms, TE = 3.872 ms, FA = 15°, and number of excitation (NEX) = 1.

The DWIs were conducted with the following parameters: TR/TE = 5700 ms/81.5 ms, FA = 90°, FOV = 100 × 100 mm^2^, matrix size = 256 × 256, 25 axial slices, voxel size = 0.94 × 0.94 × 5 mm^3^, spacing between slices = 6 mm, and NEX = 2.

### 2.3 Preprocessing of rs-fMRI

Preprocessing of rs-fMRI is to remove or at least reduce undesirable artifacts, noise, timing deviation, or spatial distortions, as well as to transform the original data to a standard format. It is essential before subjecting raw rs-fMRI data to further processing and analysis. To fulfill the functions of preprocessing, several software packages can be utilized, such as Statistical Parametric Mapping 8 (SPM8) (https://www.fil.ion.ucl.ac.uk/spm/), Resting-State fMRI Data Analysis Toolkit (REST) (https://restfmri.net/forum/rest) (Song, 2011), and Data Processing Assistant for Resting-State fMRI (DPARSF) (https://rfmri.org/DPARSF). The preprocessing comprises multiple steps: (1) discarding the first 10 rs-fMRI scans to retrospectively accommodate the time for magnetization equilibrium and the subjects’ adaptation to the environment; (2) slice-timing correction to realign all slices in time to correct the time disparity in the multi-slice acquisition; (3) six-parameter rigid-body transformation to compensate for the head motion during acquisition, by which<1-mm translation and<1° angular rotation were attained in the resultant images; (4) co-registering the T1w image to the average rs-fMRI; (5) gray matter segmentation on T1w to help construct the deformation matrix for spatial normalization of the T1w image to a Montreal Neurological Institute space; (6) utilizing the deformation matrix to conduct spatial normalization of T1w and rs-fMRI followed by a resampling with a voxel size of 3 × 3 × 3 mm^3^; (7) spatial smoothing using a Gaussian spatial filter with a 4-mm full width at half maximum (FWHM); (8) filtering with a 0.01–0.08 Hz passband to eliminate high-frequency noise as well as to detrend; (9) removal of spurious signals irrelevant to neural activities by regressing the nuisance covariates, including the six head-motion parameters, global mean signal, white matter signal, and cerebrospinal fluid signal.

### 2.4 CPM procedure

**Fig. 2:**
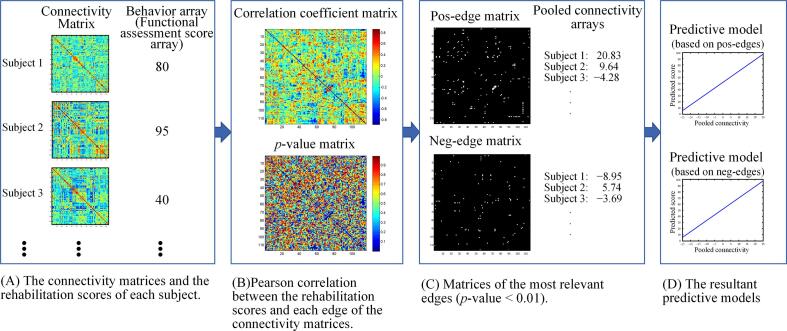
The procedure of the connectome-based predictive modeling (CPM).

Fig. 2 depicts the procedure for building predictive models based on the brain connectivity of *N* subjects. As shown in Fig. 2(A), a 2-D connectivity matrix of size *M* × *M* is created for each subject, where *M* is the number of brain regions, i.e., nodes. An element *c_ij_* of a connectivity matrix is the functional connectivity between node *i* and node *j* of the subject, which is calculated by the REST toolbox of Matlab (The MathWorks, Inc., Natick, Massachusetts, United States) from the BOLD signals of the two nodes.

The behavior array, i.e., the array of the functional assessment scores as shown in Fig. 2(A), is an *N* × 1 array. An element *b_k_* of the behavior array is the functional assessment score of subject *k*. The options for the functional assessment score in this research included mRS and BI.

The correlation coefficient matrix and the *p*-value matrix, as shown in Fig. 2(B), of size *M* × *M* represent the strength of relationship between the brain connectivity and the functional assessment score. An element *r_ij_* in the correlation coefficient matrix is the Pearson correlation coefficient between the functional assessment score and the connectivity between node *i* and node *j* over all the subjects. An element *p*_ij_ of the *p*-value matrix is the result of a significance test on the correlation coefficient *r_ij_*.

As shown in Fig. 2(C), two binary matrices of size *M* × *M*, called the pos-edge and neg-edge matrix, respectively, are built according to the content of the correlation coefficients matrix and the *p*-value matrix. The pos-edge matrix has the value 1 at each element whose corresponding edge has *r_ij_* > 0 and *p_ij_* ≤ *α*, where *α* stands for the significance level. Conversely, the neg-edge matrix has the value 1 at each element whose corresponding edge has *r_ij_* < 0 and *p_ij_* ≤ *α*. The elements of the pos-edge matrix or the neg-edge matrix corresponding to the edges with *p_ij_* > *α* are all assigned the value 0. In other words, the pos-edge and neg-edge matrices, respectively, label the edges with connectivity positively and negatively correlated significantly with the functional assessment scores.

The pooled connectivity array at pos-edges and the pooled connectivity array at neg-edges are both of size *N* × 1. An element s_k_ of the former is the inner product of the pos-edge matrix and the connectivity matrix of subject k. Similarly, an element *s_k_* of the latter is the inner product of the neg-edge matrix and the connectivity matrix of subject *k*. In other words, the value of *s_k_* is the sum of the connectivity of subject *k*’s edges with significant correlation coefficients.

A linear regression modeling is conducted on the behavior array with respect to the pooled connectivity array at pos-edges. Similarly, another linear regression modeling is conducted on the behavior array with respect to the array of pooled connectivity at neg-edges. The resultant regression models, as exemplified in Fig. 2(D), can be used to predict the functional recovery outcome of a patient by using the connectivity matrix of the patient.

### 2.5 Significant models

The CPM in this research was aimed to facilitate predicting the functional assessment scores at a later stage with the inter-regional functional connectivity at an earlier stage. Possible predictions included predicting a Stage-2 score with a Stage-1 connectivity (denoted 1 → 2), predicting a Stage-3 score with a Stage-1 connectivity (denoted 1 → 3), and predicting a Stage-3 score with a Stage-2 connectivity (denoted 2 → 3). By conducting CPM separately on the 4 patient groups, 2 assessments (i.e., mRS and BI), 3 predictions (i.e., 1 → 2, 1 → 3, and 2 → 3), and 2 edge polarities (i.e., pos-edge and neg-edge), totally 4 × 2 × 3 × 2 = 48 predictive models were generated, as will be shown in Table 3. However, not all the predictive models thus generated can be used. Analysis of the statistical significance of the correlation between the real values and the predicted values of a functional assessment score was done to validate the predictive models. Table 3 shows the results of the leave-one-out cross validation. Predictive models that lead to statistically significant correlation, i.e., with a relatively high correlation coefficient and a *p*-value lower than 0.05, would be considered significant.

### 2.6 Graphical representation

By using the BrainNet Viewer program (State Key Laboratory of Cognitive Neuroscience and Learning, Beijing Normal University, Beijing, China), the functional connectomes of the human brain can be represented with a ball-and-stick graph, where a ball represents a brain region and a stick represents the connectivity between two brain regions (Xia et al., 2013). In this study, a ball-and-stick brain net contained the edges that are considered relatively more relevant to the predictive models. In the cross-validation process for a certain predictive model, different edge patterns in pos-edge and neg-edge matrices were generated. We selected just a few edges that appeared relatively more times than most other edges in these different edge patterns. These selected edges constituted the ball-and-stick brain net shown in this paper.

## 3 Result

### 3.1 Demographics

Totally 55 out of the initially recruited 84 patients remained for the CPM procedure, screened through the three stages with the exclusion criteria shown in Fig. 1. Table 1 shows their demographics. The stroke sites of these patients were confirmed to be in the supratentorial region or the brainstem. In this research, CPM were conducted separately on the four patient groups as shown in Fig. 1. Group ST included all the 38 patients with supratentorial strokes. This group was further divided into two subgroups Group ST.L and Group ST.R that contained the 26 patients and the 12 patients whose stroke sites were in the left and right hemispheres, respectively. Group BS contained the 17 patients with brainstem strokes.

**Table 1:** Demographics of the recruited subjects.

Group	Stroke site	Subject number	Age (mean ± std)	Male/Female	Infarct volume, mL,Median (Q1, Q3)
ST	Supratentorial	38	58.59 ± 13.06	25/13	2.45 (1.45, 7.32)
ST.L	Supratentorial, left hemisphere	26	58.32 ± 17.39	18/8	2.45 (1.40, 7.32)
ST.R	Supratentorial, right hemisphere	12	54.33 ± 10.79	7/5	2.86 (1.60, 9.74)
BS	Brainstem	17	60.18 ± 9.62	10/7	0.85 (0.68, 1.22)

Table 2 summarizes the median functional assessment scores of each patient groups. This table also shows the paired comparison of individual functional assessment scores between different stages by using the Wilcoxon signed rank test. A low *p*-value in this table implies that the functional assessment scores between the two compared stages were significantly different. The progress of the functional assessment scores from Stage 1 to Stage 2 is faster than that from Stage 2 to Stage 3, as can be seen from the stage-to-stage changes of the functional assessment scores shown in Fig. 3. In this figure, the red line segments connect the average scores between two consecutive stages.

**Table 2:** The statistics for the two functional assessment scores (mRS and BI) of the patients.

Patient group	Measure	Score at Stage 1	Score at Stage 2	Score at Stage 3	*p*-value (1st vs 2nd)	*p*-value (1st vs 3rd)	*p*-value (2nd vs 3rd)
ST	mRS	2 (2,4)	2 (1,3.75)	1 (1,2)	< 0.01	< 0.01	< 0.01
	BI	60 (46.25,85)	90 (61.25,100)	100 (80,100)	< 0.01	< 0.01	< 0.01
ST.L	mRS	3(2,1)	2(1,3)	1(1,2)	< 0.01	< 0.01	< 0.01
	BI	67.5(46.25,83.75)	90(65,100)	100(87.5,100)	< 0.01	< 0.01	< 0.01
ST.R	mRS	4(2,4)	2.5(1,3)	1(1,3)	< 0.01	< 0.01	0.016
	BI	60(45,85)	90(62.5,98.75)	100(80,100)	< 0.01	< 0.01	< 0.01
BS	mRS	2.75(2.75,4)	1(1,3)	1(1,2)	< 0.01	< 0.01	0.125
	BI	43.75(43.75,85)	76.25(76.25,100)	100(91.25,100)	< 0.01	< 0.01	0.063

Note: The functional assessment scores are expressed as the median with the first and the third quartiles in the parentheses.

Note: Stage 1 = stroke onset; Stage 2 = one month after stroke onset; Stage 3 = three months after stroke onset.

**Table 3:** Correlation analysis to find significant predictive models.

Group	Measure	Prediction	With pos-edge matrix	With neg-edge matrix
			(R, *p*-value)	(R, *p*-value)
ST	mRS	1 → 2	(0.2866, 0.0902)	(0.3925, 0.0179)* 0.0078#
		1 → 3	(−0.2090, 0.2212)	(0.3746, 0.0244)
		2 → 3	(0.1188, 0.5035)	(0.2050, 0.2447)
	BI	1 → 2	(0.3157, 0.0607)	(0.0967, 0.5746)
		1 → 3	(0.4451, 0.0065)* 0.0055#	(−0.0209, 0.9038)
		2 → 3	(0.2391, 0.1732)	(−0.2716, 0.1203)
ST.L	mRS	1 → 2	(−0.2020, 0.3439)	(−0.1046, 0.6266)
		1 → 3	(−0.4158, 0.0433)	(−0.3014, 0.1523)
		2 → 3	(−0.2856, 0.1866)	(0.0435, 0.8439)
	BI	1 → 2	(−0.1021, 0.6350)	(−0.3521, 0.0915)
		1 → 3	(0.2629, 0.2146)	(−0.06, 0.7808)
		2 → 3	(0.2069, 0.3436)	(−0.5329, 0.0088)
ST.R	mRS	1 → 2	(−0.2422, 0.4483)	(−0.0207, 0.9490)
		1 → 3	(0.1735, 0.5898)	(0.3788, 0.2247)
		2 → 3	(0.6633, 0.0261)* 0.0116#	(0.1432, 0.6745)
	BI	1 → 2	(−0.0715, 0.8252)	(0.2270, 0.478)
		1 → 3	(−0.4085, 0.1874)	(−0.1573, 0.6254)
		2 → 3	(−0.3664, 0.2677)	(−0.7259, 0.0114)
BS	mRS	1 → 2	(−0.3600, 0.1558)	(−0.5943, 0.0119)
		1 → 3	(−0.8911, < 0.0001)	(−0.5213, 0.0559)
		2 → 3	(−0.4788, 0.0979)	(−0.4788, 0.0979)
	BI	1 → 2	(−0.1243, 0.6346)	(0.3490, 0.1698)
		1 → 3	(−0.2576, 0.3739)	(−0.1995, 0.4941)
		2 → 3	(−0.5284, 0.0634)	(−0.8279, 0.0005)

*Note*: i → j means to predict a Stage-j score with a Stage-i connectivity.

* indicates statistical significance, i.e. R is relatively high and *p*-value < 0.05.

# denotes the *p*-value resulting from a permutation test with 10,000 iterations.

**Fig. 3:**
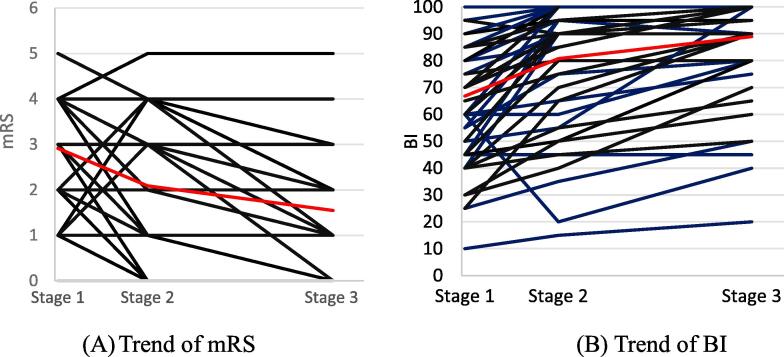
Progress of the functional assessment scores of all the subjects. The red lines link the average scores in the three stages. (mRS: modified Rankin Scale; BI: Barthel Index). (For interpretation of the references to colour in this figure legend, the reader is referred to the web version of this article.)

### 3.2 Predictive models

Shown in Table 3, the results of the leave-one-out cross validation indicate that totally 3 predictive models can be considered significant, including the 1 → 2 prediction of mRS and the 1 → 3 prediction of BI for Group ST and the 2 → 3 prediction of mRS for Group ST.R. The first one of these three models was derived based on the neg-edge matrix, while the other two were based on the pos-edge matrices. Each tuple (R, *p*-value) in Table 3 contains the Pearson's linear correlation coefficient and the *p*-value for testing the hypothesis of a non-zero correlation. Considering the unclearness of the null distribution, the permutation test was conducted for each of the three models to reconfirm their significance. The new *p*-values obtained with the permutation test were 0.0078, 0.0055, and 0.0116, which were even smaller than the original *p*-values, 0.0179, 0.0065, and 0.0261, respectively.

**Fig. 4:**
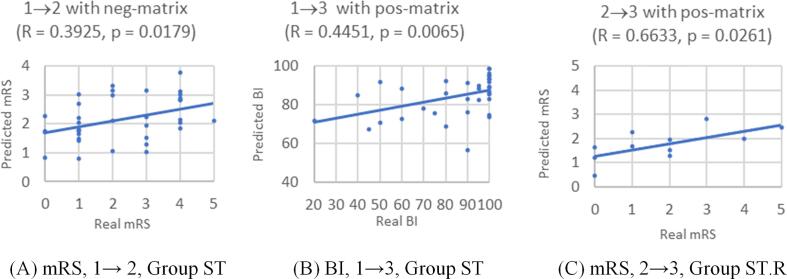
Performance of the significant predictive models. (A) Stage 1 predicting Stage 2 for mRS of Group ST; (B) Stage 1 predicting Stage 3 for BI of Group ST; (C) Stage 2 predicting Stage 3 for mRS of Group ST.R. (mRS: modified Rankin Scale; BI: Barthel Index).

Fig. 4 shows the performance of the significant predictive models by plotting the predicted values versus the true values of a functional assessment score. These models were obtained in the leave-one-out cross validation. Group ST had 38 patients, so the leave-one-out cross validation could repeat for 37 times to generate 37 predictive models. In Fig. 4(A) or (B), each of the 37 dots represents the relation between the real functional assessment score and the predictive value using one of the 37 predictive models. Fig. 4(C) contains only 11 dots because Group ST.R had 12 subjects.

### 3.3 Brain nets

**Fig. 5:**
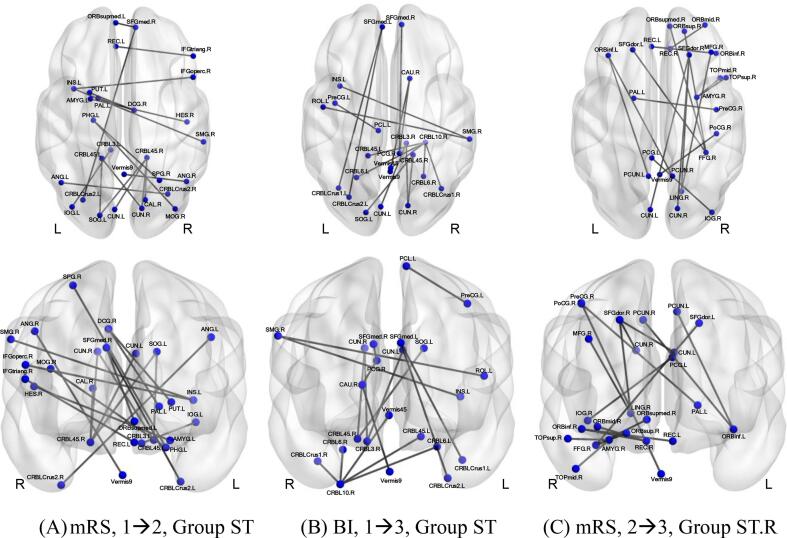
The brain nets, in the axial view (top row) and the coronal view (bottom row), corresponding to the significant predictive models. (A) Stage 1 predicting Stage 2 for mRS of Group ST; (B) Stage 1 predicting Stage 3 for BI of Group ST; (C) Stage 2 predicting Stage 3 for mRS of Group ST.R. (mRS: modified Rankin Scale; BI: Barthel Index) Abbreviations on the brain nets: AMYG = amygdala; ANG = Angular gyrus; CAL = Calcarine fissure and surrounding cortex; CAU = Caudate nucleus; CRBL = Cerebelum; CRBLCrus = Cerebelum_Crus; CUN = Cuneus; DCG = Median cingulate and paracingulate gyri; FFG = fusiform gyrus; HES = transverse temporal gyrus; IFGtriang = Inferior frontal gyrus triangular; IFGoperc = Inferior frontal gyurs opercular; INS = Insula; IOG = Inferior occipital gyrus; LING = lingual gyrus; MFG = middle frontal gyrus; MOG = Middle occipital gyrus; ORBinf = inferior frontal gyrus; ORBmid = Frontal_mid_orbital; ORBsup = medial orbitofrontal cortex; ORBsupmed = Superior frontal gyrus medial orbital; PAL = Lenticular nucleus pallidum; PCG = posterior cingulate gyrus; PCL = Paracentral lobule; PCUN = Precuneus; PHG = parahippocampal; PoCG = Postcentral gyrus; PreCG = Precentral gyrus; PUT = Lenticular nucleus putamen; REC = Gyrus rectus; ROL = Rolandic operculum; SFGdor = Superior frontal gyrus dorsolateral; SFGmed = Superior frontal gyrus, medial; SMG = Supramarginal gyrus; SOG = Superior occipital gyrus; SPG = Superior parietal gyrus; TOPmid = middle temporal pole; TOPsup = superior temporal pole.

In Fig. 5, brain nets were graphed for the acceptable (significant) prediction models. The balls and sticks are plotted with respect to the brain surface. The top row and the bottom row in this figure are the axial view and the coronal view, respectively.

## 4 Discussion

### 4.1 Function connectivity vs motor function

The ability to predict the possible course or outcome of brain functions is critical in the prognosis of poststroke care. Golestani et al. (Golestani, 2013) found that patients with impaired motor functions had lower functional connectivity derived from resting-state fMRI, and the connectivity was reestablished if the patients recovered several days afterwards. Carter et al. (Carter et al., 2012) observed contribution of decreased interhemispheric connectivity to neuromotor impairment after stroke. Puig et al. (Puig, 2018) found that incorporating functional connectivity in their prediction of 90-day modified Rankin Scale would considerably increase the accuracy than only using the 3-day National Institutes of Health Stroke Scale score. Min et al. (Min, 2020) found that the patients with better functional outcomes have greater functional connectivity. Using functional connectivity based on resting-state fMRI is advantageous in that it requires less patient cooperation and avoids the influence by the difference in types of impairment or patients’ task performance. In addition, resting-state fMRI facilitates the study of multiple networks (Golestani, 2013, Carter et al., 2012).

### 4.2 CPM

Previously, CPM has been used to predict or interpret brain functions or disorders. For example, it has been used to predict sustained attention ability (Fountain-Zaragoza, 2019) and even changes of attention in minutes, days, weeks, or months (Rosenberg, 2020). Yip et al. demonstrated the prediction of cocaine abstinence in the study of treatment outcomes of cocaine use disorders (Yip, 2019). Other than the above-mentioned, this research applies CPM for predictive modeling for poststroke functional recovery, inspired by some related research findings. For example, researchers have found association of functional recovery with preserved functional connectivity (Almeida, 2017) and decreased or increased connectivity in the contralesional hemisphere of stroke brains (De Bruyn, et al., 2021).

Brain functions are supported by widely distributed networks across brain regions. Hence, even if a stroke only causes local structural damage, dysfunction in remote regions may occur through network connections (Carter et al., 2012). For example, a lesion in the area containing corticospinal tract not only can cause direct dysfunction but also can indirectly impair upstream function by changing the network connectivity (Carter, 2012). The CPM method constructs predictive models considering all the connections in the whole brain to incorporate the effects from all parts of the brain. It is more advantageous than a prediction method based on clinical variables, which only reflect limited partial effects of the intricate brain connections.

Table 2 shows that there were significant differences between the behavior scores at Stages 1, 2, and 3. As can be seen from Fig. 3, the evolution of the scores was divergent among the patients. In other words, the recovery of different patients progressed differently. Hence, to predict a behavior score at a later stage with a behavior score at a previous stage would be inaccurate. In contrast, with the significant connectome-based predictive models among the candidates in Table 3, the brain connectivity instead of a behavior score was used to predict a behavior score at a later stage. This could result in more accurate prediction because the CPM method in this research picked predictive models consisting of the brain edges most relevant to the change of the behavior score.

As depicted in Fig. 2, not only the models based on pos-edges but also those based on neg-edges could be selected as significant predictive models. Considering that the connectivity of a neg-edge negatively correlated with the functional assessment scores, it might seem unreasonable to use a neg-edge–based predictive model to predict a functional assessment score. In fact, this can be explained by the compensational mechanism of poststroke brain behaviors. After stroke onset, the connectivity of some other edges would be strengthened to compensate the edges that were impaired by the stroke attack. As the impaired edges gradually recover, the compensating edges will also recover (i.e., revert to their original connectivity) and this leads to negative correlations between the connectivity of the compensating edges and the functional assessment score. This hypothetical theory justifies the neg-edge–based predictive models; however, further researches may be needed to verify it.

As can be seen in Fig. 3, the progress of the rehabilitation scores from Stage 1 to Stage 2 was quite different from that from Stage 2 to Stage 3. This could imply that the 1 → 2 predictability was different from the 2 → 3 predictability. Thus, for each patient group, significant models for 1 → 2 prediction and for 2 → 3 prediction didn’t necessarily concur.

The CPM method seems to possess different predictive ability for mRS and BI. This study found two significant predictive models for mRS, whereas only one for BI. Both mRS and BI are the most widely used functional outcome measures of neurological deficits. MRS is used to assess the overall disability or dependence of the patient’s daily activities. BI is used to assess the patient’s independence from physical or verbal help in feeding, bathing, grooming, dressing, toilet use, transfers, walking, and stair climbing, and degrees of bowel and bladder control. BI is prevalently used for longitudinal follow-up poststroke, whereas there is a timing effect on the sensitivity in differentiating functional recovery with the mRS (Cioncoloni, 2012). Due to mRS–BI discrepancies in the target behavior functions and the timing effects, that a significant model for mRS can be found does not ensure that a significant model for BI can also be found, and vice versa.

A predictive model constructed with the CPM method takes the inter-regional connectivity computed from the rs-fMRI signals to predict the functional recovery. The modeling and predicting is readily automated and can be harmoniously integrated into the modern intelligent clinical procedure.

### 4.3 Brain nets

The brain nets shown in Fig. 5 contain balls and sticks that represent, respectively, the brain regions and the connectivity between regions. With 116 brain regions involved in the CPM procedure, the maximum number of edges in a brain net would be (116 × 116 – 116)/2 = 6670. Each brain net shown in Fig. 5 contains only about 20 edged, shown as the sticks, among the possible 6670 edges. These selected edges dominated the basis on which the predictive models were derived. The brain regions connected by these dominant edges in a brain net were supposed to be more relevant to the attributes of the functional assessment score.

As seen in Fig. 5, the dominant regions seemed to be distributed in a widespread brain territory, not reflecting the infarct lesions’ localness, concentration characteristic. This phenomenon would seem reasonable if we consider that the connections affected by stroke are not only limited to the vicinity of the infarcted lesion but also between remote regions in the ipsilesional and the contralesional hemispheres (Rehme and Grefkes, 2013) and a stroke can compromise multiple brain functions not explainable by the focal damage alone (Dørum, 2020).

The brain net in Fig. 5(B) is for predicting BI of Group ST patients. The dominant nodes in this brain net were more concentrated in the posterior fossa, which is related to balancing and coordination of motor functions. Balancing and coordination are important factors affecting the score of BI. Hence, it is reasonable that a brain net for predicting BI has more dominant nodes in the posterior fossa.

The brain net in Fig. 5(C) was for predicting mRS of Group ST.R. The dominant nodes in this brain net were mostly in the right hemisphere. This phenomenon corresponds with the fact that the patients in Group ST.R had supratentorial infarction in only the right hemisphere. This phenomenon was not found in the brain net in Fig. 5(A) for predicting mRS of Group ST patients or the brain net in Fig. R(B) for predicting BI of Group ST patients, corresponding with the fact that Group ST patients had infarction in both the right and left hemispheres. Hence, the dominant nodes tend to appear in the infarcted hemisphere.

Group BS comprised patients with infarction in the brain stem, which has complex connection to cerebellum and neurons of cranial nerves besides corticospinal tracts. This could imply that Group BS requires predictive models beyond the complexity of the CPM developed in this research. This might explain why no significant predictive model was found in this research for predicting mRS or BI for Group BS.

Limitations.

There were some limitations of this research.

The first limitation was due to the mRS score. The recruited patients were limited to those with mRS between 2 and 4 points. The resultant predictive models would not be applicable to patients that score 0, 1, or 5 points in mRS.

The second limitation was due to the sample sizes. The sample sizes of the four patient groups were 38, 26, 12 and 17. With such sample sizes, it could not be affordable to divide the patients into subgroups for analyzing some confounding factors such as age, gender, infarct size and location, and NIHSS. Besides, small sample sizes might hinder the generation of significant predictive models. This was probably the reason why only 3 were significant among the 24 predictive models that were generated in the CPM procedure in this research. Possibly more significant predictive models could be generated if there had been more patients involved in the CPM procedure. In addition, the four patient groups consisted of imbalanced numbers of different genders. The gender bias might reduce the generalizability of the significant predictive models. Moreover, a small sample size could result in overfitted predicted models. An overfitted connectome-based predictive model would contain more than adequate number of edges to account for the behavior of the population. The extra edges would reflect the peculiarity of the data used in the training procedure, i.e., the model-building procedure shown in Fig. 2. To alleviate the effect of small sample sizes, the leave-one-out cross validation was adopted.

The third limitation was due to the discrepancy in the assessment dates. The nominal assessment dates were specified as the day of stroke onset, one month and three months after stroke onset, respectively, for Stage 1, 2, and 3. However, the real assessment dates were 0–10 days, one month ± 7 days and three months ± 7 days after stroke onset, as indicated in Fig. 1. This discrepancy in the assessment dates among different patients could be a source of prediction error.

## 5 Conclusion

This research demonstrated that the connectome-based predictive models for predicting functional recovery outcome of acute ischemic stroke can be constructed with the CPM method. In this research, using the CPM method, 48 models were developed for predicting the functional assessment scores from the functional connectivity of a previous stage. Among these prediction models, three were found significant. For the patients with supratentorial strokes, one model was found significant for predicting BI at the third stage with brain connectivity at the first stage, and another one was found significant for predicting mRS at the second stage with the brain connectivity at the first stage. In the patients with right hemispheric supratentorial strokes, one model was found significant for predicting mRS at the third stage with the brain connectivity at the second stage. For the patients with brainstem strokes, none of the predictive models was significant.

From observing these models, we found that the significant model for predicting BI contained more nodes in the posterior fossa, whose function is related to balance and coordination. The significant model for predicting mRS reveals that more significant nodes tended to be distributed in the ipsilesional hemisphere.

In summary, the CPM method is a potential tool for constructing connectome-based models for predicting the outcome of poststroke functional recovery. This method is evident-based and can be easily automated. However, the results of this research were based on small samples and hence they might not be readily applicable clinically. Provided that the predictive models are derived from and crucially validated with large samples, the predicted functional assessment scores could possibly serve as objective bases in clinical prognosis.

## CRediT authorship contribution statement

**Syu-Jyun Peng:** Conceptualization, Methodology, Software, Validation, Formal analysis, Data curation, Supervision. **Yu-Wei Chen:** Conceptualization, Validation, Investigation, Resources, Supervision, Project administration, Funding acquisition. **Andrew Hung:** Software, Formal analysis, Investigation, Data curation, Writing – original draft, Visualization. **Kuo-Wei Wang:** Resources, Funding acquisition. **Jang-Zern Tsai:** Conceptualization, Methodology, Investigation, Writing – review & editing, Supervision, Project administration, Funding acquisition.

## Declaration of Competing Interest

The authors declare that they have no known competing financial interests or personal relationships that could have appeared to influence the work reported in this paper.

## Data Availability

The authors do not have permission to share data.
